# Novel *Escherichia coli* phages representing a distinct genus within the subfamily *Stephanstirmvirinae*: genome and host range characteristics

**DOI:** 10.1007/s00705-025-06469-1

**Published:** 2025-12-10

**Authors:** Tomoyoshi Kaneko, Jumpei Uchiyama, Toshifumi Osaka, Satoshi Tsuneda

**Affiliations:** 1 Department of Life Science and Medical Bioscience, Waseda University, Tokyo, Japan; 2 Phage Therapy Institute, Comprehensive Research Organization, Waseda University, Tokyo, Japan; 3 Department of Bacteriology, Graduate School of Medicine Dentistry and Pharmaceutical Sciences, Okayama University, Okayama, Japan; 4 Department of Microbiology and Immunology, Tokyo Women’s Medical University, Tokyo, Japan

## Abstract

Bacteriophages play crucial roles in microbial ecosystems and have potential for biotechnological applications. However, our understanding of culturable phages remains limited. In this study, we characterized six novel *Escherichia coli* phages isolated from pig farm wastewater and urban sewage, using comprehensive genomic, morphological, and host-range analysis. Using multiple comparative approaches, including gene-sharing network analysis, average nucleotide identity (ANI), and nucleotide intergenomic similarity (NIS), we demonstrated that five of these phages form a distinct group within the subfamily *Stephanstirmvirinae*, potentially representing a novel genus, for which we propose the name "*Wecvirus*”. We further propose that these phages, each of which exhibits a unique host range pattern, should be classified in two distinct species within the proposed genus. This host specificity is reflected in differences in the amino acid sequences of tail fibers, which are crucial for infection. The remaining phage, which was not classified as a wecvirus, exhibited characteristics that challenged the current classification criteria, highlighting the need for more-flexible taxonomic approaches. These findings expand our understanding of phage diversity within the subfamily *Stephanstirmvirinae* and contribute to the evolving phage taxonomy framework.

**Supplementary Information:**

The online version contains supplementary material available at 10.1007/s00705-025-06469-1.

## Introduction

Bacteriophages (phages) are viruses that infect bacteria and are the most abundant biological entities on Earth [1]. They play vital roles in shaping microbial communities, driving bacterial evolution, and maintaining the ecological balance in various environments [2–5]. The diversity and abundance of phages significantly influence global biogeochemical cycles and hold promise for biotechnological applications, including phage therapy and biocontrol [6–8].

Phage classification is challenging because of the rapid evolution and extensive genetic diversity of phages [9]. The International Committee on Taxonomy of Viruses (ICTV) has continually updated its classification system for viruses, including phages, to accommodate new discoveries and genomic data. In 2022, the ICTV substantially revised the classification of members of the former order "*Caudovirales*", abandoning the morphology-based classification in favor of a system based on genetic relationships [10].

The subfamily *Stephanstirmvirinae*, a relatively new taxonomic group within the class *Caudoviricetes*, consists of two genera: *Justusliebigvirus* and *Phapecoctavirus*. Phages in this subfamily primarily infect gammaproteobacteria, including *Escherichia coli*, *Klebsiella pneumoniae*, and members of the *Pseudomonadales* [11–17]. Interestingly, *Campylobacter jejuni*, an epsilonproteobacterium with a distinct outer membrane structure, has also been reported to be a host for members of the *Stephanstirmvirinae*, suggesting that phages in this subfamily may possess diverse host recognition mechanisms [18]. These bacterial hosts are medically significant because of their rapid development of antimicrobial resistance, and phages infecting these bacteria are considered promising candidates for combating drug-resistant pathogens [6, 7, 19]. These phages have been isolated from diverse environments, including water samples (sewage and freshwater), feces (avian and human), and compost, suggesting their ability to infect bacterial strains from different sources [11–13, 18]. Phages belonging to the genera *Justusliebigvirus* and *Phapecoctavirus* have been reported to show lytic activity against drug-resistant bacteria, human pathogens, and plant pathogens, leading to ongoing research into their potential as novel antimicrobial agents [13–15, 17]. The diversity of host recognition mechanisms in these phages indicates their potential to adapt to different bacterial strains. This characteristic, combined with their demonstrated activity against various pathogenic bacteria, makes these phages promising candidates for therapeutic applications. Understanding these host-phage interactions is crucial for developing effective phage-based treatments against emerging drug-resistant bacteria.

As of August 29, 2024, eight publications on “*Phapecoctavirus*” and one on “*Justusliebigvirus*” have been indexed in PubMed [11–18, 20]. While these studies have established basic characteristics, such as double-stranded DNA genomes (~ 150 kbp) and GC content (~ 39%), many critical aspects remain unexplored. In particular, the mechanisms underlying host specificity, including the role of structural proteins, such as tail fibers, warrant further investigation to understand their evolutionary adaptations and therapeutic potential.

In this study, we present a comprehensive characterization of six phage isolates: four previously isolated from pig farm wastewater (ΦWec179, ΦWec181, ΦWec186, and ΦWec187) and two from urban sewage (ΦWec188 and ΦWec190) [21]. Our analysis included a phylogenetic analysis based on genome sequences, morphological features observed using transmission electron microscopy (TEM), and host range evaluation. This study expands our understanding of phage diversity within the subfamily *Stephanstirmvirinae* and contributes significantly to the broader field of phage taxonomy and evolution.

## Materials and methods

### Reagents and bacteria

Luria-Bertani medium was used for bacterial and phage cultures. The concentration of soft agar was 0.5%. The *E. coli* strain TK001 used in this study was isolated from the feces of a mouse with dextran sodium sulfate (DSS)-induced colitis and was used as the host strain for phage screening [21]. The animal experiments for generating mice with DSS-induced colitis used for the isolation of TK001 were reviewed and approved by the Waseda University Academic Research Ethics Committee (approval number 2020-A009).

### Phage preparation

Four *E. coli* phages (ΦWec179, 181, 186, and 187) were isolated from swine farm effluent, and two phages (ΦWec188 and 190) were isolated from municipal sewage. These six phages were propagated using *E. coli* TK001 as the host, while phages T4 and T7 were propagated using MG1655 as the host, all using the plate lysate method. Briefly, an overnight bacterial culture (100 µL) and phage suspension (10^4^ PFU/mL, 100 µL) were added to soft agar (0.5%) and overlaid onto agar plates. After overnight incubation (37°C), 5 mL of sodium chloride–magnesium sulfate (SM) buffer was added to the plates, followed by orbital shaking (approximately 250 rpm, 2–3 h). The supernatant was collected in 15-mL tubes and centrifuged at 7,300 *g* for 15 min at 4 °C in a TOMY MX-305 centrifuge with an AR500-03 rotor to separate the solid and liquid components. The liquid component was transferred to new 15-mL tubes, followed by the addition of 1 mL of chloroform. After vortexing and settling, the preparation was used as the phage stock. Phages T4 and T7 were obtained from the Biological Resource Center of the National Institute of Technology and Evaluation in Kisarazu, Japan (NBRC 20004 and 20005, respectively).

### Host range analysis

The ability of the phages to infect various bacterial strains was evaluated as follows: Each phage (approximately 10^7^ PFU/mL) was serially diluted tenfold up to a 10^7^ dilution in SM buffer, 1 µL of which was spotted onto a bacterial lawn. The efficiency of plating (EoP) was calculated by dividing the titer against each bacterial strain by the titer against the reference host (TK001 for ΦWec179, 181, 186, 188, and 190; MG1655 for T4 and T7). The bacterial strains tested included 33 laboratory and clinical isolates of *E. coli*, four different serotypes of *Salmonella enterica* (NBRC3163, 3313, 13245, and 15335), and one strain of *E. fergusonii* (NBRC102419), details of which are provided in Supplementary Table S1. Serotype determination of *E. coli* strains was performed using SerotypeFinder 2.0 [22].

### TEM

Phages (10^9^−10^10^ PFU/mL) were purified via polyethylene glycol precipitation and loaded onto copper grids using a support film (cat. no. 649; Nissin EM Co. Ltd., Tokyo, Japan). After washing three times with distilled water, the samples were stained with fourfold-diluted EM Stainer (cat. no. 336; Nissin EM Co., Ltd., Tokyo, Japan) for 1 min. The excess liquid was removed with filter paper, and the grids were air-dried before observation using a JEM-1010 transmission electron microscope (JEOL, Tokyo, Japan) at 100 kV.

### Taxonomic analysis based on whole genome sequences

Previously published genome sequences (LC739533-LC739537 and LC739539) were used [21]. Gene-sharing network analysis was performed using vConTACT2 with reference genome sequences of 3,503 phages (ProkaryoticViralRefSeq201) and visualized using Cytoscape v3.10.1 [23, 24]. Taxonomic information that was not updated within vConTACT2 was updated manually using a taxonomy browser (https://www.ncbi.nlm.nih.gov/Taxonomy/Browser/wwwtax.cgi). ANI analysis was performed using pyani with the "anib" parameter [25]. NIS was calculated using VIRIDIC [26]. The phage genomes used for the ANI and NIS comparisons were obtained using the Genbank_get_genomes_by_taxon.py script included in the Pyani package. Comparative genome analysis was performed using Genomematcher in blastp mode [27]. Synteny visualization between representative phages and type strains from related genera was performed using Clinker v0.0.31[28].

### Genome annotation

Phage annotations were performed using Pharokka to complement previous annotations performed using RAST and DFAST [29]. A phylogenetic tree was constructed by the maximum-likelihood method with 1,000 bootstrap replicates, using MEGA 11 [30]. Provisional naming conventions were used to facilitate comparison of the tail-fiber-protein-coding genes. Based on the number of amino acid residues encoded, the genes were designated as follows: TfpL (943 or 958 residues), TfpM1 (890 or 891 residues), TfpM2 (681 residues), TfpS1 (353 residues), TfpS2 (235 residues), and TfpS3 (157 residues). These designations are used for discussion purposes in this report and are not proposed as official gene names.

## Results

### Morphological analysis

**Fig. 1:**
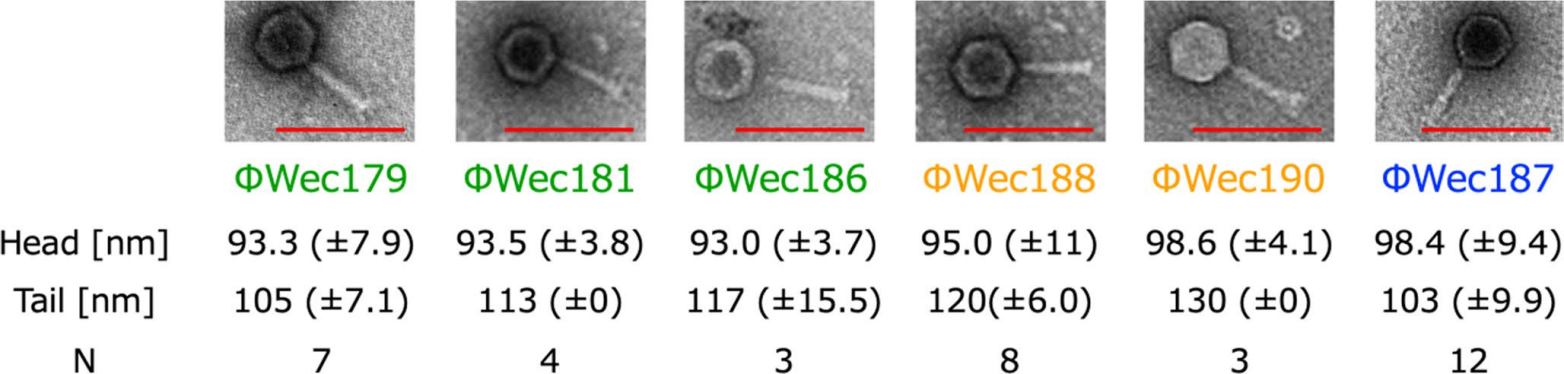
TEM images of phages. Representative TEM images of each phage with mean values and standard deviations for head and tail size. The scale bar represents 200 nm

The TEM images showed that all of the phages were morphologically identical to myoviruses (Fig. 1), and all of their capsids and tails exhibited similar morphology.

### Host range analysis

**Fig. 2:**
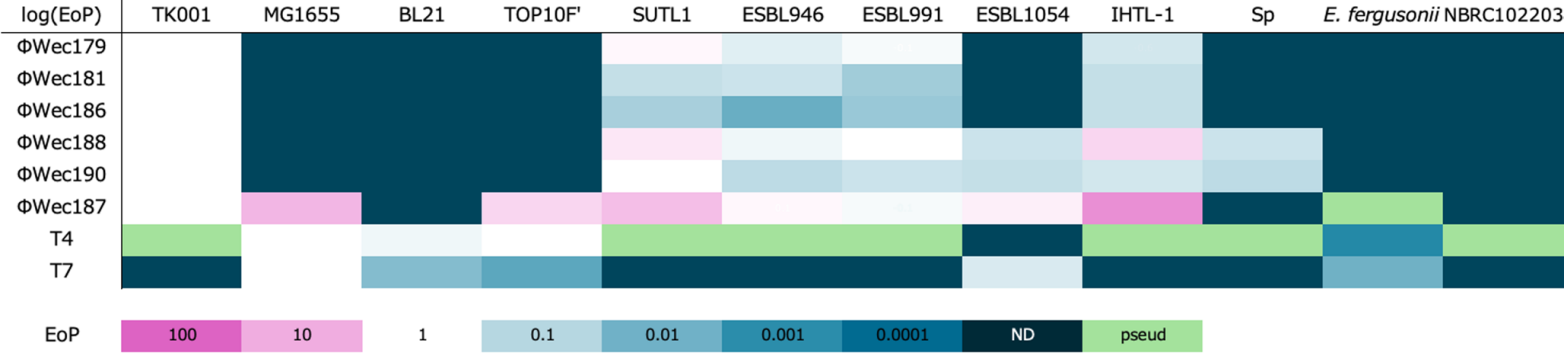
Heat map of the host range of the isolated phages. Host range analysis was performed by spot assays with serial dilutions of each phage on various bacterial strains. The EoP (efficiency of plating) was calculated by dividing the titer against each bacterial strain by the titer against the reference host strain (TK001 for ΦWec179, 181, 186, 188, and 190; MG1655 for T4 and T7). White indicates no reduction in infectivity (EoP = 1), and progressively darker cyan shades represent decreasing infectivity levels, with dark cyan signifying complete loss of infectivity (not detected, ND). Purple shades indicate enhanced infectivity (EoP > 1). "pseud" indicates that plaques were not visible but lysis was observed in the bacterial lawn. "ND" indicates that no lysis or plaque formation occurred even at the highest phage concentration tested

Host range analysis with multiple *Enterobacteriaceae* species revealed that these phages showed similar patterns, all infecting *E. coli* TK001 and the *E. coli* clinical isolates SUTL1, ESBL946, and ESBL991 (Fig. 2 and Supplementary Fig. S1). The ΦWec188 and 190 phages could also infect ESBL1054 and *S. enterica* subsp. *enterica* serovar Pullorum. Furthermore, ΦWec187 could infect all of the strains that were susceptible to the other phages plus ESBL1054 and the *E. coli* laboratory strains MG1655 and TOP10F', and it showed lytic activity (without plaque formation) against *E. fergusonii*. The control phages T4 and T7 infected three laboratory strains (MG1655, TOP10F', and BL21) and *E. fergusonii*, with T7 also infecting the clinical isolate ESBL1054. Serotype determination of *E. coli* strains was performed, but no clear correlation was observed between phage infectivity patterns and bacterial serotypes, suggesting that host specificity is determined by factors other than serotype-specific determinants (Supplementary Table S1).

### Taxonomic analysis based on whole genome sequences

Our isolates possess a typical myovirus genome organization with genes arranged in functional clusters: early genes involved in DNA replication and recombination, followed by structural genes encoding capsid and tail proteins, and late genes for lysis functions. All of the genomes contain more than 10 tRNA genes and terminate with direct terminal repeats, like other *Stephanstirmvirinae* members.

BLASTn analysis revealed that among the six phages characterized, ΦWec179, 181, and 186 showed the highest BLAST ANI (identity × coverage) to *Escherichia* phage vB_EcoM_DE16 (accession no. OP595145.1) of the genus *Phapecoctavirus* within the subfamily *Stephanstirmvirinae*, with a BLAST ANI of 55.32% (80.18% identity × 69% coverage). ΦWec187, which showed notably lower GC content (37.5%) than the other phage isolates (42.2–42.3%), was most closely related to *Escherichia* phage AV123 (accession no. OR352953.1) of the genus *Justusliebigvirus* with a BLAST ANI of 94.17% (98.09% identity × 96% coverage). ΦWec188 and 190 showed the highest BLAST ANI to *Escherichia* phage Mt1B1_P17 (accession no. NC_052662.1) of the genus *Phapecoctavirus*, with a BLAST ANI of 54.36% (77.66% identity × 70% coverage) (Supplementary Table S2). The genomic characteristics of the phages are summarized in Supplementary Figure S2.

**Fig. 3:**
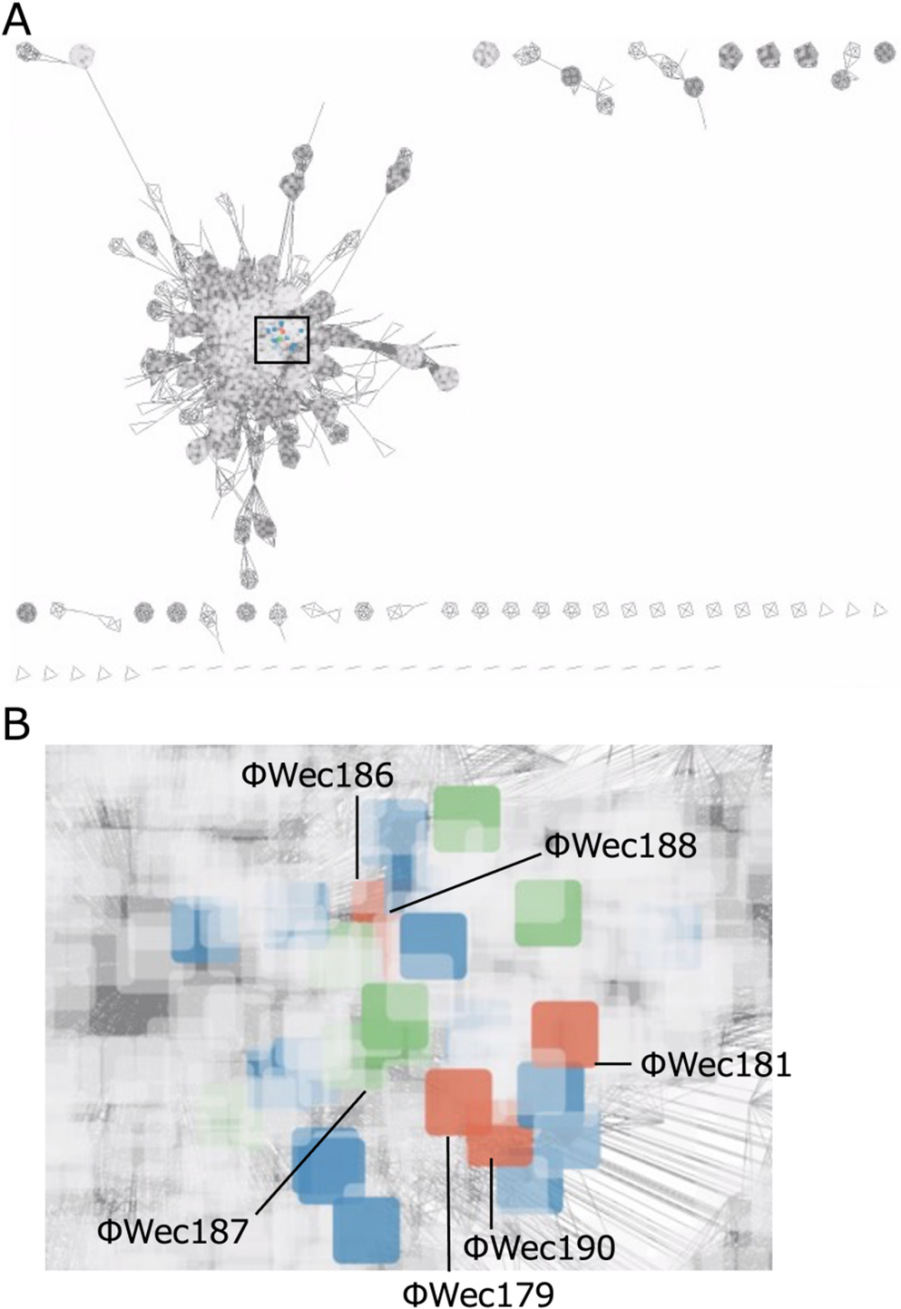
Gene-sharing network analysis of phage genomes. (**A**) Network visualization of gene-sharing relationships among 3,509 phage genomes supplemented with 15 additional *Stephanstirmvirinae* members (10 *Phapecoctavirus* and five *Justusliebigvirus* phages) not included in the original reference dataset to ensure complete representation of these genera. The boxed area indicates the VC315 cluster. (**B**) Enlarged view of the VC315 cluster showing subclusters: VC315_0 (green node) contains ΦWec187 and reference phages belonging to *Justusliebigvirus*, VC315_1 (orange nodes) consists of ΦWec179, 181, 186, 188, and 190, VC315_2 (light blue node) contains reference phages belonging to *Phapecoctavirus*, and gray nodes indicate other reference phages.

To investigate the relationships between these phage isolates more thoroughly, we performed a gene-sharing network analysis based on shared protein clusters between viral genomes using vContact2, including all known members of the genera *Justusliebigvirus* and *Phapecoctavirus* (Fig. 3A, Supplementary Table S3). ΦWec179, 181, 186, 188, and 190 formed their own unique cluster (VC315_1) with ΦWec179, 181, and 186 clustering closely together, while ΦWec188 and 190 formed a separate group within the same cluster (Fig. 3B, Supplementary Table S3). ΦWec187 belonged to VC315_0, which contained all members of the genus *Justusliebigvirus* of the subfamily *Stephanstirmvirinae* (Fig. 3B, Supplementary Table S3). VC315_2 comprised all members of the genus *Phapecoctavirus* of the subfamily *Stephanstirmvirinae*. This clustering pattern demonstrates that all known members of each established genus grouped together, while our isolates formed distinct clusters.

**Fig. 4:**
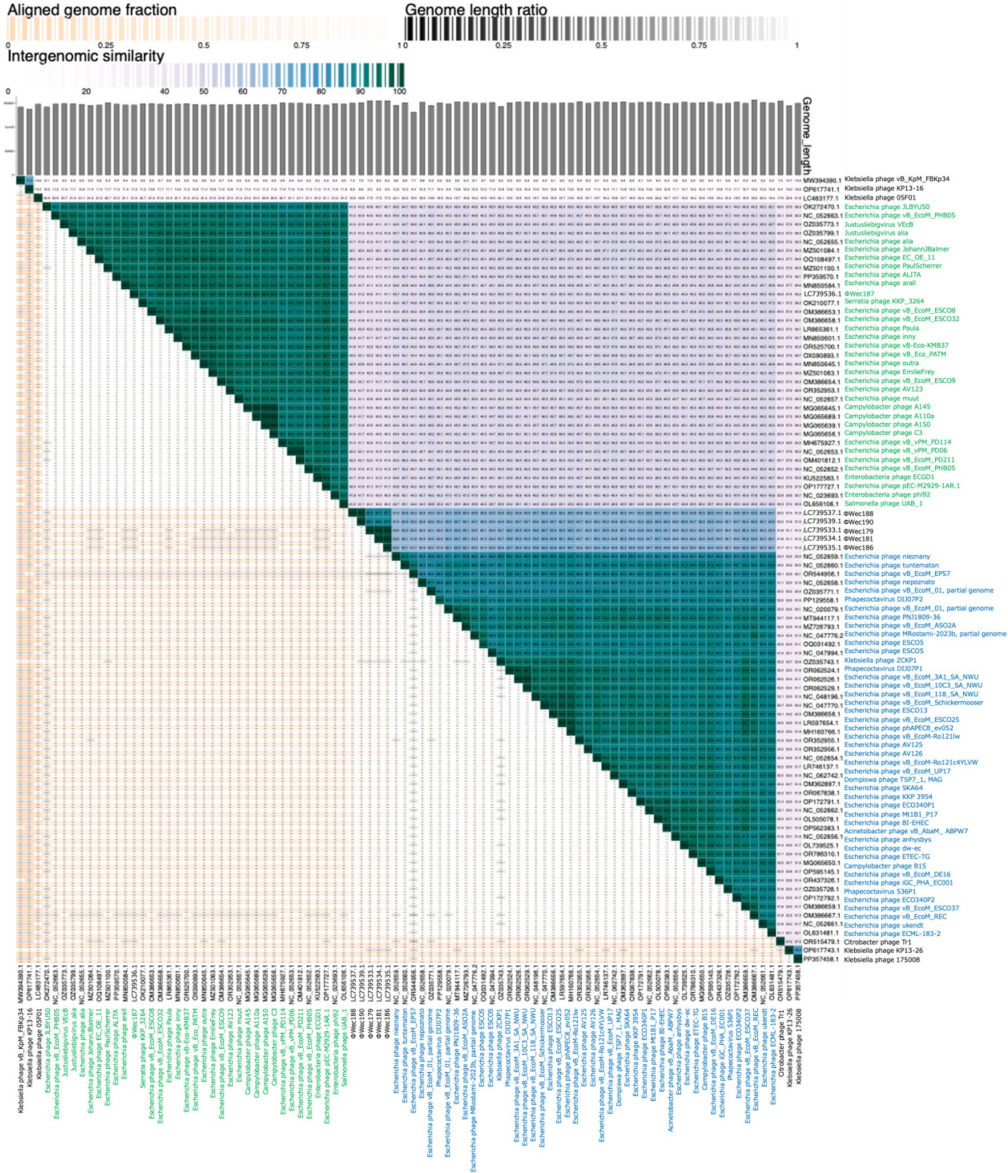
Similarity analysis of phage isolates of the subfamily *Stephanstirmvirinae*. NIS, genome length, and aligned genome fractions were determined using VIRIDIC. The phage genome information for comparison was obtained from registered *Stephanstirmvirinae* members using the genbank_get_genomes_by_taxon.py script included in the pyani package. Phage names are provided alongside accession numbers for clarity. Green shading indicates *Justusliebigvirus* members, and blue shading indicates *Phapecoctavirus* members.

To evaluate the similarity between all NCBI-registered *Stephanstirmvirinae* subfamily phages (as of July 24, 2024) and ΦWec179, 181, 186, 187, 188, and 190, we calculated the nucleotide intergenomic similarity (NIS) using VIRIDIC and average nucleotide identity (ANI) using pyani (Fig. 4, Supplementary Table S4). ΦWec187 showed NIS values below 95.5% when compared to the registered *Stephanstirmvirinae* phages, whereas its ANI values were between 0.7% and 94%. Notably, Campylobacter phage A145 (MG065645.1), Campylobacter phage A110a (MG065689.1), Campylobacter phage A150 (MG065639.1), Campylobacter phage C3 (MG065656.1), Escherichia phage vB_Eco_PATM (OX090893.1), and Escherichia phage Paula (LR865361.1) showed NIS values between 95% and 95.5% with ΦWec187, and their ANI values were approximately 90%. However, some phages with ANI values above 90% had NIS values below 95%, whereas others with ANI values below 90% (approximately 89%) had NIS values above 95%. ΦWec179, 181, 186, 188, and 190 showed NIS values below 66% and ANI values between 20 and 50% when compared to *Stephanstirmvirinae* members registered in NCBI. Furthermore, both NIS and ANI values for ΦWec179, 181, 186, 188, and 190 exceeded 70%; they formed two distinct groups – the ΦWec179, 181, and 186 and ΦWec188 and 190 groups – when a 90% threshold was applied.

Comparative genomic analysis visually confirmed the high similarity suggested by NIS and ANI values within the ΦWec179, 181, and 186 and ΦWec188 and 190 groups (Supplementary Fig. S2A and B). Comparison between ΦWec179 and ΦWec188 revealed some similarity, although it was less pronounced than that within groups (Supplementary Fig. S2C). Comparison between representative strains ΦWec179 (from the ΦWec179, 181, and 186 group), ΦWec188 (from the ΦWec188, 190 group), and ΦWec187 showed negligible similarity.

**Fig. 5:**
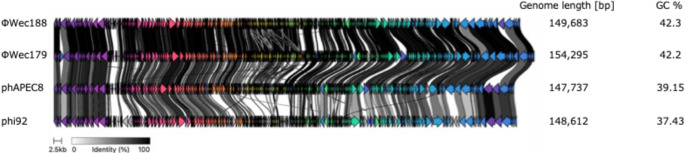
Comparative genomic analysis of representative phages with type strains from related genera. Comparative genome visualization of phiWec188, phiWec179, phAPEC8 (*Phapecoctavirus* type strain), and phi92 (*Justusliebigvirus* type strain). Arrows represent ORFs, and connecting ribbons indicate homologous regions with shading intensity reflecting sequence similarity. Genome length and GC content are shown on the right.

To further investigate the evolutionary relationships between our isolates and members of the established *Stephanstirmvirinae* genera, we performed comparative genomic analysis with type strains belonging to the genera *Phapecoctavirus* (phAPEC8) and *Justusliebigvirus* (phi92). This analysis revealed limited synteny between our isolates and the reference strains (Fig. 5). The comparative analysis identified conserved genes suitable for phylogenetic reconstruction, including the large terminase subunit, major capsid protein, and tail fiber genes.

### Phylogenetic analysis of conserved structural and tail fiber proteins

To investigate evolutionary relationships and host-interaction mechanisms, we conducted phylogenetic analysis of both conserved structural genes (major capsid protein and terminase large subunit) and tail fiber genes. Tail fiber genes were selected for analysis because they are crucial determinants of phage-host interactions through bacterial surface receptor recognition [31–33]. Six types of genes encoding tail fiber proteins were identified based on similarity to sequences in the VOG database, using the results of Pharokka annotation: TfpL (long tail fiber protein; 943 or 958 amino acids [aa]), TfpM1 (major tail fiber protein 1; 890 or 891 aa), TfpM2 (major tail fiber protein 2; 681 aa), TfpS1 (short tail fiber protein 1; 353 aa), TfpS2 (short tail fiber protein 2; 235 aa), and TfpS3 (short tail fiber protein 3; 157 aa). These genes were present in all phages except for the justusliebigviruses, including ΦWec187, which lacked the gene encoding TfpM1.tusli

**Fig. 6:**
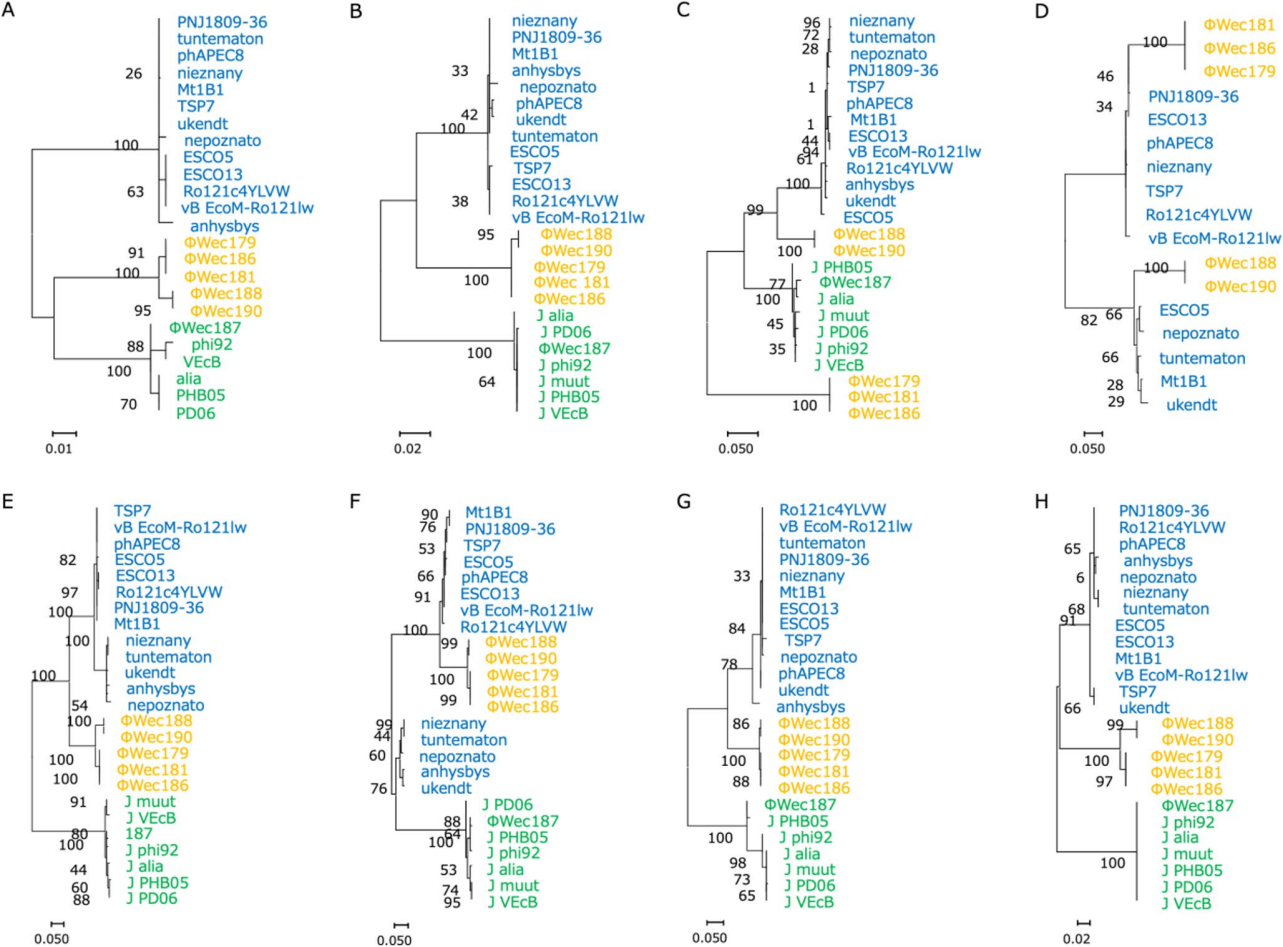
Molecular phylogenetic trees based on the amino acid sequences of tail fiber genes. Trees were constructed using the ML method with 1000 bootstrap replicates. Blue indicates *Phapecoctavirus* members, green indicates *Justusliebigvirus* members, and yellow indicates our isolates (ΦWec179, 181, 186, 188, 190). (**A**) Major head (capsid) protein. (**B**) Terminase large subunit (Escherichia phage alia excluded due to gene spanning contig boundaries). (**C**) TfpL. (**D**) TfpM1 (absent in *Justusliebigvirus* members; Escherichia phage anhysbys excluded due to gene spanning contig boundaries). (**E**) TfpM2. (**F**) TfpS1. (**G**) TfpS2. (**H**) TfpS3

Molecular phylogenetic analysis revealed distinct clustering patterns across different protein families. For structural genes (major capsid protein and terminase large subunit) and most tail fiber genes (TfpM2, TfpS2, TfpS3), our isolates (ΦWec179, 181, 186, 188, 190), *Phapecoctavirus* members, and *Justusliebigvirus* members formed clearly separated clades (Fig. 6A, B, E, G, H). However, gene-specific variations were observed: TfpS1 showed mixed clustering, with *Phapecoctavirus* forming two separate groups while our isolates and *Justusliebigvirus* clustered together (Fig. 6F). Notably, TfpL and TfpM1 revealed internal structure within the ΦWec179, 181, 186, 188, and 190 group, with ΦWec179, 181, and 186 forming one subclade and ΦWec188 and 190 forming another, (Fig. 6C and D). TfpM1 was absent in all *Justusliebigvirus* members, indicating genus-specific differences in tail fiber composition (Fig. 6E).

Furthermore, while each gene encoding a tail fiber protein (TfpL, TfpM1, TfpM2, TfpS1, TfpS2, and TfpS3) was completely conserved within the ΦWec179 group and the ΦWec188 group, slight differences were observed between the groups, with variations concentrated in the C-terminal regions. Notably, although these genes were perfectly conserved within the ΦWec188 group, their order was not conserved. Additionally, the arrangement of conserved tail fiber genes (TfpL, TfpM2, TfpS1, TfpS2, and TfpS3) in ΦWec187 matched that in ΦWec188.

## Discussion

In this study, we characterized six novel *E. coli* phages, using genomic, morphological, and host range analysis. Our findings revealed that five of these phages (ΦWec179, ΦWec181, ΦWec186, ΦWec188, and ΦWec190) represent a new genus within the subfamily *Stephanstirmvirinae*, for which we propose the name "*Wecvirus*”. These phages form two distinct species, based on their genomic similarities and host range patterns. The remaining phage, ΦWec187, shows characteristics of a *Justusliebigvirus*, although it cannot be classified accurately under the current classification criteria.

Morphological analysis showed that all the phage isolates have a myovirus morphotype, consistent with that observed for other members of the subfamily *Stephanstirmvirinae* [12–14, 16]. Host range patterns revealed distinct infection profiles among the isolated phages. From the perspective of host range testing, all of the environmental phages analyzed in this study, including T4 (class *Caudoviricetes*, family *Straboviridae*, subfamily *Tevenvirinae*, genus *Tequatrovirus*) and T7 (class *Caudoviricetes*, family *Autotranscriptaviridae*, subfamily *Studiervirinae*, genus *Teseptimavirus*), which are phylogenetically distinct from the wecviruses, infected approximately 13% of the host strains, confirming the narrow host range typical of *Enterobacteriaceae*-infecting phages [34]. The differential ability to infect laboratory strains versus clinical isolates suggests an adaptation to specific host populations. Notably, the broader host range of ΦWec187 compared to those of other isolates indicates potential differences in host recognition mechanisms. Our findings align with those of previous studies on *Stephanstirmvirinae* members, where similar variations in host specificity were reported. For instance, Nicolas *et al*. reported that *Phapecoctavirus* phages infected approximately 24% of the strains in a panel of 32 *E. coli* strains, primarily avian pathogenic *E. coli* (APEC) strains, whereas "*Wecvirus*" isolates demonstrated a narrower host range against different clinical isolates [12]. Similarly, Markuskovál *et al*. observed that the *Justusliebigvirus* phage phi92 had a 23% infection rate against uropathogenic *E. coli* [17]. These differences in host specificity possibly reflect the diverse origins of the tested bacterial strains rather than inherent differences in host range breadth.

Taxonomic analysis revealed significant evolutionary relationships among members of the subfamily *Stephanstirmvirinae*. Phages ΦWec179, ΦWec181, ΦWec186, ΦWec188, and ΦWec190 formed a distinct cluster and showed similarity values below the 70% genus-level threshold in both NIS and ANI analysis when compared with all known members. This discovery suggests that the current sampling has not yet captured the full range of phage diversity within this subfamily. The limited synteny observed between "*Wecvirus*" and established genera (phAPEC8 and phi92) in comparative genomic analysis further supports the proposal of "*Wecvirus*" as a distinct genus within the subfamily *Stephanstirmvirinae*.

Among these five phages, NIS and ANI analysis revealed two distinct clusters with high levels of internal similarity (> 95%). Between-group similarity values of 80–88% indicated that these clusters represent separate species, despite their close evolutionary relationship. The consistently higher NIS values (60–65%) compared to the corresponding ANI values (20–50%) between these phages and reference phages reflect fundamental differences in how these metrics are calculated. These patterns were consistent with BLASTn results: ΦWec187 showed high similarity to known *Justusliebigvirus* members (BLAST ANI: 94.17%), while "*Wecvirus*" phages (ΦWec179-190) showed low similarity to known *Phapecoctavirus* members (BLAST ANI < 70%), supporting their classification as a distinct genus. NIS, which performs direct whole-genome comparisons, appears to capture conserved regions and overall genome structure more effectively. Conversely, ANI values, which represent average similarities across fragmented sequences [35], may better evaluate local similarities but do not completely account for phage-specific genomic features, such as extensive rearrangements or large-scale insertions/deletions.

We observed discrepancies between the NIS and ANI values of ΦWec187, making classification of this phage under the current taxonomic criteria challenging. The unique characteristics of this phage highlight the complexity of current phage classification systems and warrant further investigation. We propose that ΦWec187 should be temporarily classified as “unclassified *Stephanstirmvirinae*” and investigated in the future.

Phylogenetic analysis of structural genes provided additional support for our taxonomic proposals. Both major capsid protein and terminase large subunit phylogenies clearly separated our isolates from the established genera *Phapecoctavirus* and *Justusliebigvirus*, demonstrating that the distinctiveness observed in whole-genome analysis extends to conserved structural components. These results confirm that the proposed genus "*Wecvirus*" represents a genuine evolutionary lineage rather than a product of extensive horizontal gene transfer or recombination events that might affect only variable genomic regions.

Our findings regarding tail fiber diversity in the "*Wecvirus*" phages provide insights into the relationship between phage structure and host specificity within the subfamily *Stephanstirmvirinae*. Interestingly, the type strains of the two established genera in this subfamily exhibit notably broad host ranges. Phage phi92 (*Justusliebigvirus*) demonstrates remarkable versatility, which can be attributed to its four distinct types of tail fibers/tailspikes that enable attachment to various bacterial surface structures, allowing infection of both *E. coli* and *Salmonella* strains [36]. Similarly, phAPEC8 (*Phapecoctavirus*) shows broad infectivity against various APEC strains [37]. In contrast, our "*Wecvirus*" isolates demonstrated narrower host ranges (approximately 13% infectivity), which is more typical of *Enterobacteriaceae*-infecting phages.

The species-specific differences in tail fiber composition we observed in the wecviruses likely represent a more specialized evolutionary strategy for host adaptation, perhaps not as extensive as the "multivalent adsorption apparatus" described for phi92 [36]. This structural difference in tail fiber complexity may explain why "*Wecvirus*" members exhibit more-restricted host ranges than other *Stephanstirmvirinae* members, suggesting that tail fiber diversity is a key determinant of host range breadth within this subfamily.

Tail fibers are composed of multiple proteins that play crucial roles in host recognition [38, 39]. For example, the proximal and distal tail fibers of phage T4 have distinct protein compositions [40]. The multiple tail fiber genes identified in this study possibly constitute different parts of the tail structure and may enable the recognition of different host molecules [41].

Detailed analysis of tail fiber proteins provides insights into phage-host interactions and adaptation mechanisms. The differences in host ranges correlate with group-specific characteristics of tail fiber composition, particularly in the C-terminal regions, which are crucial for receptor recognition [42–45]. While whole-genome comparisons showed clear grouping patterns, phylogenetic analysis based on tail fiber gene sequences revealed more-complex relationships, particularly regarding ΦWec187, suggesting mosaic evolution via horizontal gene transfer rather than simple linear evolution [9, 46].

Our previous study demonstrated that all five phages target the R1 core lipopolysaccharide (LPS), with both groups specifically recognizing galactose transferred by WaaT [47]. The C-terminal sequence variations in tail fibers suggest the acquisition of additional host recognition capabilities while maintaining this core LPS-binding function. In particular, the ΦWec179-186 group appears to utilize flagella as a secondary receptor, while the ΦWec188-190 group requires the inner membrane protein, YhaH, indicating distinct evolutionary trajectories in host adaptation.

Subtle differences in host range were observed, even within the groups. For example, ΦWec179, 181, and 186, despite belonging to the same group and showing complete conservation of tail fiber protein sequences, exhibited slight variations in infection efficiency against certain strains. These observations suggest that, while the tail structure largely determines the host range, host utilization efficiency may involve factors beyond phage adsorption, such as replication efficiency within host cells. For instance, the T4 collar and whiskers regulate long tail fiber retraction under unfavorable conditions [48], and bacterial phage defense systems can reduce efficiency of plating (EoP) [49]. Future research should focus on the functional analysis of receptor-binding proteins and investigation of interactions with the host cell surface. Additionally, functional analysis of genes whose functions are currently unknown may contribute to our understanding of host specificity.

Based on the results of this study and per the ICTV naming conventions (Rules 3.21, 3.22) [50], we propose the following taxonomic classifications:

1.  Establishment of a new genus, “*Wecvirus*”, within the subfamily *Stephanstirmvirinae*
2.  Designation of a new species, “*Wecvirus wec179*”, comprising ΦWec179, ΦWec181, and ΦWec186
3. Designation of a new species, “*Wecvirus wec188*”, comprising ΦWec188 and ΦWec190

These names follow the ICTV guidelines [50], with the genus name consisting of a single word with the suffix “-*virus*” and the species names comprising two words: the genus name followed by the isolate number. The new genus name is based on the representative isolate designation (Wec). The members of the proposed genus "*Wecvirus*" are genetically distinct from known *Stephanstirmvirinae* subfamily members (ANI and NIS < 70%), and the members of each of its proposed species (“*Wecvirus wec179*” and “*Wecvirus wec188*”) display unique host ranges. The establishment of this new genus and its constituent species will enhance our understanding of phage diversity and refine the taxonomic system within the subfamily *Stephanstirmvirinae*. Future research is expected to identify additional phages belonging to this new genus and elucidate the ecological role of its members through functional analysis of receptor-binding proteins and investigation of host-phage interactions.

## Electronic Supplementary Material

Below is the link to the electronic supplementary material

Supplementary Material 1(XLSX 316 KB)

Supplementary Material 2(DOCX 2.46 MB)

Supplementary Material 3(XLSX 346 KB)

Supplementary Material 4(XLSX 319 KB)

Supplementary Material 5(DOCX 2.81 MB)

Supplementary Material 6(DOCX 5.17 MB)

## Data Availability

All data supporting the findings of this study are included within the manuscript and its supplementary information files.
