# Supplementary material for: Novel Escherichia coli phages representing a distinct genus within the subfamily Stephanstirmvirinae: genome and host range characteristics
Source: Arch Virol. 2025 Dec 10;171(1):18. doi: 10.1007/s00705-025-06469-1 (PMC12695924; doi:10.1007/s00705-025-06469-1)
Supplement: Supplementary file 2 — Supplementary Material 2(DOCX 2.46 MB) [file 705_2025_6469_MOESM2_ESM.docx]

**Fig. S1 Heatmap of host range of the isolated phages with all bacterial strains.** Host range analysis was performed by spot assays with serial dilutions of each phage on various bacterial strains. EoP (Efficiency of Plating) was calculated by dividing the titer against each bacterial strain by the titer against the reference host strain (TK001 for ΦWec179, 181, 186, 188, and 190; MG1655 for T4 and T7). White indicates no reduction in infectivity (EoP = 1), and progressively darker cyan shades represent decreasing infectivity levels, with dark cyan signifying complete loss of infectivity (not detected, ND). Purple shades indicate enhanced infectivity (EoP > 1). "pseud" indicates that plaques were not visible but lysis was observed in the bacterial lawn. "ND" indicates that no lysis or plaque formation occurred even at the highest phage concentration tested. For detailed examination, refer to SupplementaryTables_and_FigS1.xlsx.

**
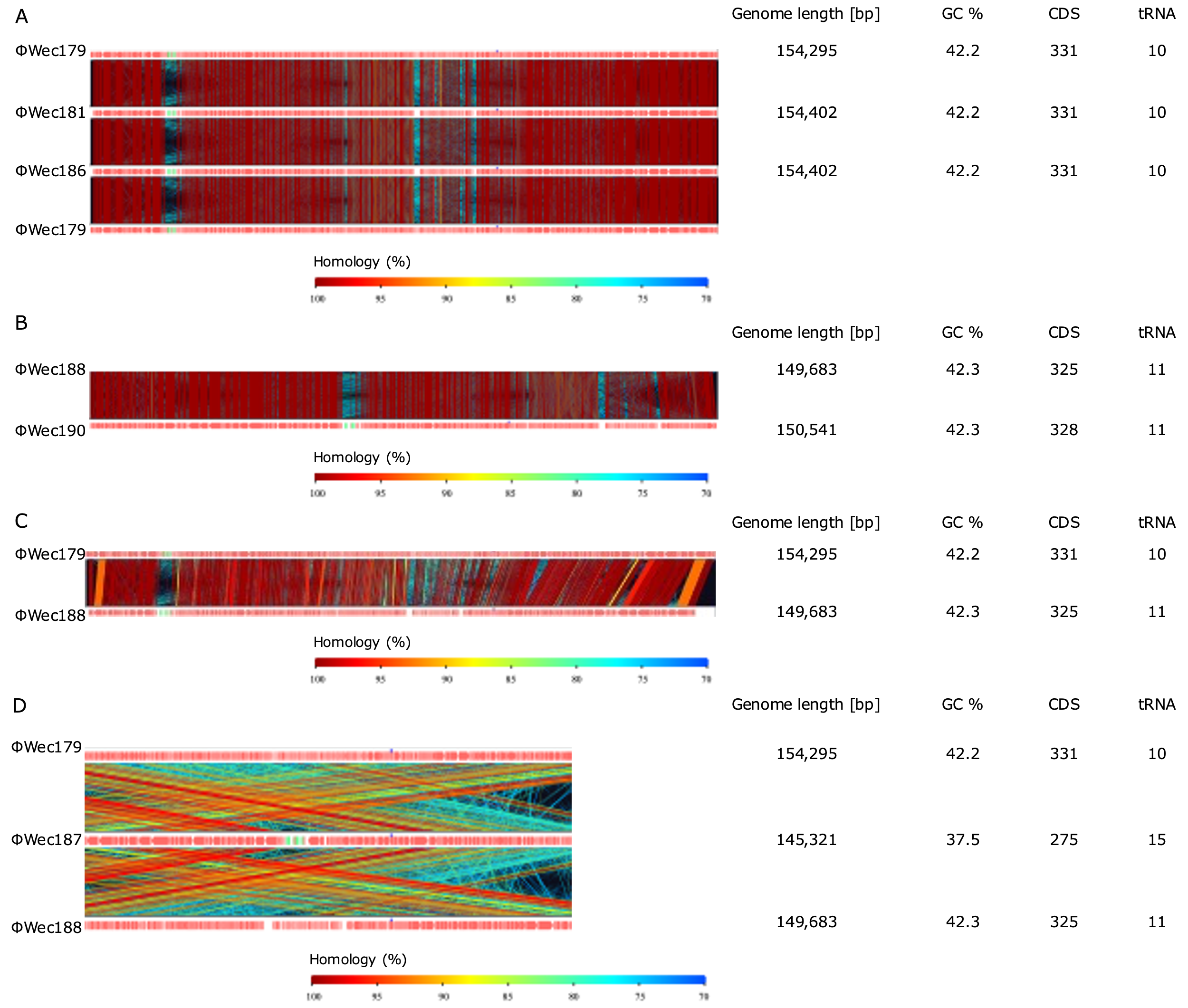
**

**Fig. S2 Comparative genomic analysis of isolated phages**. Heatmap representation of genome similarities. (A) ΦWec179, 181, and 186 group. (B) ΦWec188 and 190. (C) ΦWec179 and 188. (D) ΦWec179, 187, and 188 group. Genome length, GC content, number of CDS, and number of tRNA are shown on the right.
